# Quantitative Assessment of Microbial Transmission onto Environmental Surfaces Using Thermoresponsive Gelatin Hydrogels as a Finger Mimetic under In Situ‐Mimicking Conditions

**DOI:** 10.1002/adhm.202403790

**Published:** 2025-01-15

**Authors:** Mihyun Lee, Luzia Wiesli, Frank Schreiber, Angela Ivask, Qun Ren

**Affiliations:** ^1^ Laboratory for Biointerfaces Empa, Swiss Federal Laboratories for Materials and Technology Lerchenfeldstrasse 5 St. Gallen 9014 Switzerland; ^2^ Division of Biodeterioration and Reference Organisms (4.1) Department of Materials and the Environment Federal Institute for Materials Research and Testing (BAM) Unter den Eichen 87 12205 Berlin Germany; ^3^ Institute of Molecular and Cell Biology University of Tartu Riia 23 Tartu 51010 Estonia

**Keywords:** antimicrobial testing, high‐touch surfaces, real‐life conditions, thermoresponsive hydrogels, touch transfer assay

## Abstract

Surface‐mediated transmission of pathogens plays a key role in healthcare‐associated infections. However, proper techniques for its quantitative analysis are lacking, making it challenging to develop novel antimicrobial and anti‐fouling surfaces to reduce pathogen spread via environmental surfaces. This study demonstrates a gelatin hydrogel‐based touch transfer test, the HydroTouch test, to evaluate pathogen transmission on high‐touch surfaces under semi‐dry conditions. The HydroTouch test employs gelatin as a finger mimetic, facilitating testing with pathogenic bacteria under controlled conditions. The thermoresponsive sol–gel transition of gelatin allows easy recovery and quantification of bacteria before and after testing. The HydroTouch test demonstrates that methicillin‐resistant *Staphylococcus aureus* has a high transmission efficiency of ≈16% onto stainless steel, compared to <3% for *Escherichia coli* or *Pseudomonas aeruginosa*. Polyurethane surfaces exhibit strong resistance to bacterial contamination with a transmission efficiency of ≈0.6%, while polytetrafluoroethylene shows a transmission efficiency approximately four times higher than polyurethane. Additionally, quaternary ammonium‐based antimicrobial coatings reduce the transmission efficiency of live bacteria on stainless steel to ≈4% of the original level. The HydroTouch test provides a reliable method for assessing pathogen transmission on various surfaces under semi‐dry settings, supporting the development of effective antimicrobial, anti‐transmission coatings to reduce healthcare‐associated infections.

## Graphical abstract

A novel method for the quantitative assessment of pathogenic bacteria transmission is established using thermoresponsive gelatin as a finger‐mimetic. The HydroTouch test reveals that methicillin‐resistant *S. aureus* is highly transferable onto stainless steel compared to *E. coli* and *P. aeruginosa*. Quaternary ammonium‐based antimicrobial coatings on stainless steel efficiently reduce microbial transmission efficiency under conditions closely mimicking real‐life scenarios.

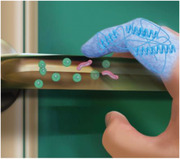

## 1 Introduction

Recent studies have highlighted the critical role of environmental surfaces in the transmission of pathogens as a major mechanism of microbial spread.^[^ ^1^, ^2^ ^]^ Notably, healthcare‐associated infections (HAIs) are significantly influenced by surface‐mediated transmission, which has been estimated to cause ≈20–40% of HAIs.^[^ ^3^ ^]^ Pathogens can contaminate the surfaces of medical equipment and high‐touch surfaces such as door handles, tables, and nurse‐call buttons followed by potential transmission to healthcare personnel and finally, to patients. Several key pathogens including methicillin‐resistant *Staphylococcus aureus* (MRSA) and vancomycin‐resistant *Enterococcus* (VRE) have been shown to survive on environmental surfaces for extended periods, increasing the risk of their transmission.^[^ ^4^ ^]^ A number of studies have demonstrated that effective disinfection of environmental surfaces can significantly reduce surface‐mediated infections.^[^ ^5^, ^6^ ^]^ However, maintaining optimal disinfection practices consistently turned out to be a practical challenge.^[^ ^7^ ^]^ As a complementary strategy, products with intrinsic antimicrobial properties are frequently used. Copper, silver, and quaternary ammonium‐based surface coatings are the most common materials due to their high antimicrobial efficiency via release and/or contact‐killing mechanisms.^[^ ^8^, ^9^ ^]^ However, maintaining long‐term continuous antimicrobial and/or antifouling activities remains challenging, in addition to the potential cytotoxic issues.

For efficient prevention and reduction of surface‐mediated transmission of live microbial pathogens an ideal surface should not only avoid microbial adhesion but also kill microbes that adhere to it. The development of such surfaces necessitates a proper assessment of microbial transmission. However, standard methods enable primarily the assessment of the killing effect of a given surface. Furthermore, common antimicrobial tests, such as ISO 22196^[^ ^10^ ^]^ and ISO 7581,^[^ ^11^ ^]^ are conducted under completely wet or dry conditions neither of which mimics the semi‐dry environment of finger skin. However, different moisture conditions can significantly impact the measured antimicrobial efficacy of different surfaces.^[^ ^12^, ^13^, ^14^, ^15^ ^]^ Evaluation of transmission efficiency can be conducted apart from antimicrobial testing, often using human fingers. Typically, volunteers are trained to press on a scale to achieve a pre‐defined weight load for a certain time.^[^ ^16^ ^]^ The use of human fingers offers realistic semi‐dry conditions but introduces high variability due to differences between individuals, making it difficult to achieve reliable results. Additionally, using human fingers poses safety concerns, preventing the testing of potentially pathogenic bacteria. As alternatives, methods involving a stamp made of synthetic materials such as silicon rubber and textile instead of a finger have been devised.^[^ ^17^, ^18^ ^]^ For example, bacteria were loaded onto the surface of polydimethylsiloxane (PDMS), which was then pressed onto a test surface, with a weight immediately placed on top to control the load.^[^ ^18^ ^]^ This method allows for controlled load application and testing of pathogenic microbes. However, the surface composition of PDMS differs greatly from real skin, impacting interactions between the surface and microbes, and therefore microbial attachment. Additionally, the dry environment of PDMS can cause microbial death due to desiccation, complicating the quantification of live microbes transmitted to the surface.^[^ ^19^, ^20^ ^]^

**Figure 1:**
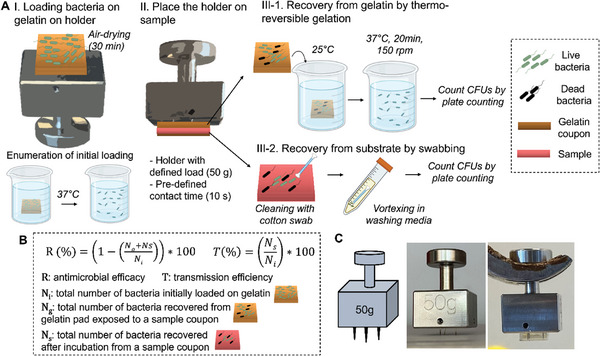
Design of the HydroTouch test. A) Experimental procedure for the touch transfer test. The gelatin pad is installed on the holder followed by loading with bacterial inoculum and drying for a defined time. For enumeration of initial loading, the gelatin pad is dissolved at 37 °C without contacting the coupons, followed by CFU quantification (I). For transfer testing, the gelatin pad on the holder is placed on the coupon for a defined contact time (II). Afterward, the bacteria on the gelatin pad are recovered via thermoresponsive sol–gel transition (III‐1), and the tested coupon is cleaned by swabbing to collect bacteria (III‐2). Colony‐forming units (CFU) are counted by plate counting. B) Formulas to calculate antimicrobial efficacy and transmission efficiency of live bacteria in the touch transfer method. C) Design of the holder (left, middle) and the holder with a gelatin pad (right).

Herein, a testing method is developed utilizing a hydrogel to evaluate pathogen transmission and antimicrobial efficacy of surfaces under realistic conditions. This method, named the HydroTouch test, allows us to predict the transmission of living pathogens onto surfaces under conditions that closely mimic real‐life scenarios, where a microbially contaminated finger touches a surface. The method utilizes a thermoresponsive gelatin hydrogel, which replicates several key characteristics of the human finger. The surface of the gelatin hydrogel provides a semi‐dry environment similar to that of human finger surfaces, reducing the risk of desiccation‐induced bacterial cell death compared to a completely dry surface. Gelatin is produced through the hydrolysis of collagen, the most abundant protein in human skin.^[^ ^21^ ^]^ Due to its low cost, high biocompatibility, and favorable mechanical properties, gelatin has been extensively utilized in various fields, including biomedical engineering, pharmaceuticals, and food engineering.^[^ ^22^ ^]^ In particular, gelatin forms thermoresponsive, physically crosslinked hydrogels, where triple helix structures are induced upon an increase in temperature. This property makes gelatin one of the most popular materials for 3D bioprinting to produce human tissues. In the HydroTouch test, gelatin not only mimics the wettability, chemical composition, and semi‐dry nature of a human finger, but also ensures reliable and complete recovery of bacteria for quantification through thermoresponsive sol–gel transition. Additionally, to achieve a controlled weight load for a defined period, a metallic holder is designed as shown in **Figure** **1**. Using the HydroTouch test, the transmission of different bacterial strains including MRSA, *E. coli*, and *P. aeruginosa* on stainless steel is investigated. Furthermore, the transmission of *E. coli* onto various plastics, including polyurethane and polytetrafluoroethylene, and to two antimicrobial surfaces, copper and quaternary ammonium coated on stainless steel, is investigated. Overall, the HydroTouch test provides a reliable and quantitative approach to studying pathogen transmission on high‐touch surfaces, supporting the development of more effective antimicrobial coatings and infection control strategies.

## 2 Results

### 2.1 Establishment of the HydroTouch Test

The HydroTouch test procedure is illustrated in Figure 1A. A gelatin pad is mounted on a metal holder, and a defined number of bacteria are loaded onto the gelatin pad (Figure 1A‐I). The holder is then placed on a coupon for a pre‐defined duration (e.g. 10 s) (Figure 1A‐II). Subsequently, the gelatin pad is transferred to a washing medium and incubated at 37 °C to induce a transition to sol, allowing for the quantification of colony‐forming units (CFUs) by plate counting (Figure 1A‐III‐1). The coupon is incubated in the air for a defined period, after which the bacteria are washed off using a cotton swab, followed by CFU enumeration by plate counting (Figure 1‐III‐2). The initial bacterial loading on the gelatin is quantified by counting CFUs recovered from gelatin without contacting coupon surfaces (Figure 1‐I). The antimicrobial efficacy and transmission efficiency of live bacteria are calculated using the equations provided in Figure 1B. Transmission efficiency focuses on the fraction of viable cells present on the coupon surface after a short contact and a defined incubation time on the coupon. This fraction therefore includes the combined effects of the ability of the surface to receive cells transferred from the gelatin pad (the finger mimetic) and the antimicrobial effect exerted by the surface. The calculated antimicrobial efficacy for a coupon accounts for its effect on cells residing on the gelatin during the short contact and on the cells transferred to the surface.

**Figure 2:**
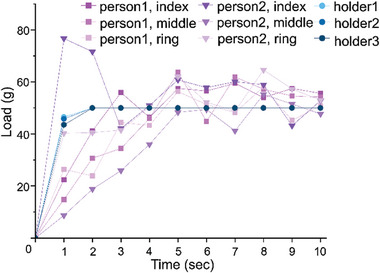
Stability of weight loading for 10s by human fingers and metal holders.

A metallic holder was designed to enable the loading of a defined weight on a coupon and to stably hold a gelatin pad with three needles in the middle (Figure 1C, left). The holder was fabricated using stainless steel (Figure 1C, middle), and designed such that a gelatin pad (11 mm × 11 mm × 3 mm; width × length × height) was stably held by the holder (Figure 1C, right). The weight of the holder was chosen to be 50 g, which is considered comparable to the load of a light finger touch in everyday life.^[^ ^23^, ^24^ ^]^ The stability of weight loading by the holder was compared with the weight loading by the fingers of two volunteers, who were trained to load 50 g on a scale for 10 s. As shown in **Figure** **2**, the use of the holder enabled reaching 50 g within 2 s and maintaining this load constantly until the end of the given time. In contrast, with human fingers, it took 4 to 5 s to approach 50 g, and the weight fluctuated throughout the entire testing period. In particular, the sudden decrease in load observed for person 2 between 2 and 3 s shows the unreliable nature of loading caused by human error.

**Figure 3:**
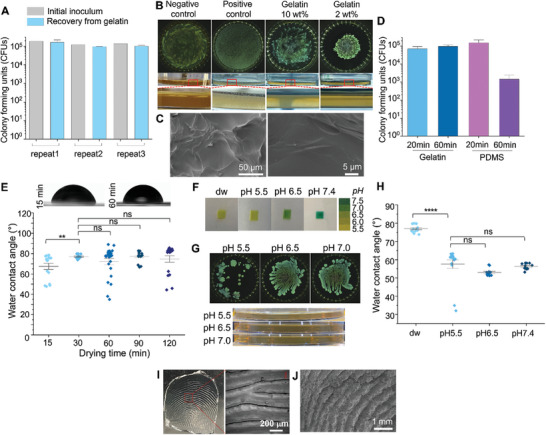
Suitability of gelatin for HydroTouch test. A) CFU quantified for the initial inoculum of *E. coli* and for the recovered bacteria from gelatin via thermoresponsive sol–gel transition. B) *E. coli* grown on gelatin gels in a Petri dish (d = 90 mm). Top: Top view. Middle: Side view. Bottom: Enlarged side view. C) SEM images of gelatin gels. D) Bacterial recovery from gelatin via thermoresponsive sol transition and from PDMS via vortexing after drying for 20 min or 60 min. E) Water contact angle (WCA) of gelatin gel according to drying time. F) pH indicator within gelatin gels prepared using water (dw) or buffer solutions. G) *E. coli* grown on gelatin gels at various pH ranges in a Petri dish (d = 90 mm). Top: Top view. Bottom: Side view. H) WCA of gelatin gel according to pH. I, J) Gelatin gel with fingerprint structures observed by optical microscopy (I) and SEM (J). Error bars represent the standard deviation generated from 3 replicates in an experiment. Data was collected from three repeat experiments for A and one experiment for D, E, and H. ^**^: *p* ≤ 0.01, ^****^: *p* ≤ 0.0001, ns: *p* ≥ 0.05.

The suitability of gelatin gels for touch transfer testing was assessed. To evaluate the reliability of loading and unloading bacteria on gelatin, a bacterial suspension was applied to the surface of the gelatin pad. The gelatin was then dissolved by increasing the temperature to 37 °C. The recovered cell concentration was ≈80% of the initially loaded number, still within the 5‐log range, indicating that unloading bacteria via the thermoresponsive sol–gel transition of gelatin is effective (**Figure** **3**A).

For the HydroTouch test, ensuring that bacteria do not penetrate the gelatin gel is crucial. To test this, suspensions of gram‐negative (*E. coli*) or gram‐positive (*S. epidermis* and *L. lactis*) bacteria were spread on the gelatin gel and kept at room temperature (RT) overnight. To support bacterial growth, gelatin gels containing culture media (LB for *E. coli*, BHI for *S. epidermidis*, and *L. lactis*) were used for this test. As shown in Figure 3B (gram‐negative) and Figure S1 (Supporting Information) (gram‐positive), bacterial colonies were only found on the surface of the gelatin pad, at both 10 and 2 wt% gelatin gels, similar to the negative control, the agar gel. If bacteria had penetrated the gel, bacterial colonies would have been visible, as seen in the positive control, which was prepared by mixing bacteria with warm (≈37 °C) gelatin solution before gelation. The resistance of the gelatin gel to bacterial penetration was attributed to its non‐porous surface structure, as observed by SEM (Figure 3C). Next, bacterial recovery from a gelatin pad was compared with that from PDMS, a material previously used as a stamp for touch transfer tests.^[^ ^18^ ^]^ For testing, a bacterial suspension was applied to a gelatin pad or PDMS surface, and dried for either 20 or 60 min. Subsequently, bacteria were recovered from a gelatin pad via thermoresponsive sol–gel transition, whereas for PDMS swabbing was used to collect bacteria from the surface. The recovery rate of bacteria from gelatin after 20 min of drying was comparable to that measured for PDMS (Figure 3D). Interestingly, the recovery rate from gelatin was not affected by the tested drying time, whereas recovery from PDMS significantly decreased after 60 min of drying compared to 20 min. The swabbed PDMS coupons were cultivated under an agar gel to assess residual bacteria on the surface. Colonies were detected on all three coupons dried for 30 min. However, for the coupons dried for 60 min, only one out of three showed colony growth. (Figure S2, Supporting Information) To determine if the physicochemical properties of gelatin pad surfaces are suitable as a finger mimetic, its surface wettability, pH adjustability, and tenability of microscale structures to mimic fingerprints were investigated. The water contact angle (WCA) of gelatin gel after drying at RT was measured (Figure 3E). After 15 min of drying, the WCA was 67.4 ± 12.2°, stabilizing to 77.0 ± 2.1° after 30 min, falling within the reported range for human fingers.^[^ ^25^ ^]^ In addition, the gelatin gels were successfully prepared at pH 5.5, pH 6.5, and pH 7.4 (Figure 3F) and no bacterial penetration was confirmed for any of the gels (Figure 3G). The WCA decreased to ≈60° for pads between pH 5.5 to 7.4 as compared to the pad prepared in water, remaining within the range of human finger pH. (Figure 3H). Moreover, it was possible to generate fingerprint‐like structures on gelatin using a negative template made from a human finger, with a resolution of 100–500 µm on gelatin (Figure 3I,J).

**Figure 4:**
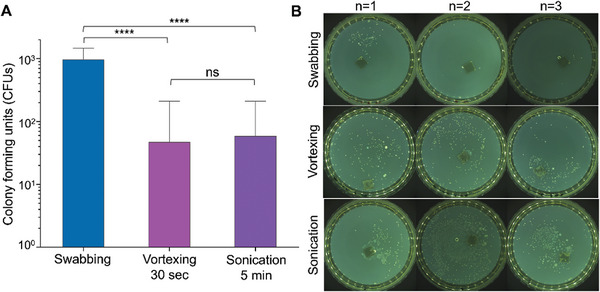
Optimization of the method for bacterial recovery from tested coupons. A) CFUs recovered from stainless steel after a HydroTouch test using different recovery methods. Error bars represent the standard deviation generated from 3 replicates in one experiment. ^****^: *p* ≤ 0.0001, ns: *p* ≥ 0.05. B) Residual bacteria on stainless steel after washing were grown as colonies within agar gels. An agar solution at ≈40 °C was poured onto washed SS, followed by gelation and incubation at 37 °C overnight.

To optimize bacterial recovery from tested coupons, three washing methods—swabbing, vortexing, and sonication—were compared using stainless steel as a model high‐touch coupon. Approximately 10 times more CFUs were recovered by swabbing compared to vortexing and sonication (**Figure** **4**A). Additionally, the swabbing method showed less variation between replicates compared to the other methods. The residual bacteria on the swab‐cleaned coupons were found to be negligible (Figure 4B).

### 2.2 Transmission of Bacteria onto Stainless Steel

**Figure 5:**
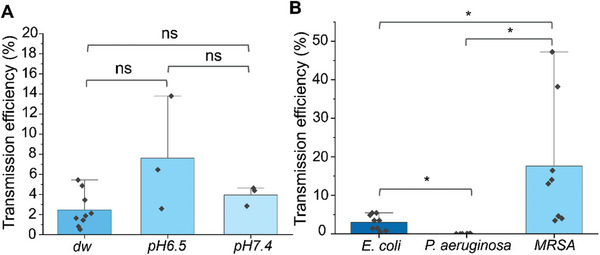
Transmission of pathogenic bacteria onto stainless steel. A) Transmission of *E. coli* onto stainless steel depending on pH of gelatin gels. B) Transmission of *E. coli, P. aeruginosa*, and *MRSA* onto stainless steel. Error bars represent the standard deviation generated from 9 replicates in three experiments for dw in A and B, and 3 replicates in one experiment for the rest. ^*^: *p* ≤ 0.05, ns: *p* ≥ 0.05.

The transmission of *E. coli* onto stainless steel coupons was investigated using the established HydroTouch method. The impact of pH on transmission was first examined using gelatin pads prepared with deionized water (dw) (≈pH 5.5) and buffer solutions at pH 6.5 or pH 7.4. Results indicated that pH levels within the tested range do not significantly influence the transmission of *E. coli* (**Figure** **5**A). Next, the transmission of pathogenic bacteria onto stainless steel was studied, using two Gram‐negative strains, *E. coli* and *P. aeruginosa*, and a drug‐resistant Gram‐positive bacterium, *MRSA* (Figure 5B). Interestingly, MRSA demonstrated a much higher transmission efficiency compared to the two Gram‐negative pathogens, with *P. aeruginosa* showing a very low transmission efficiency.

### 2.3 Transmission of Bacteria onto Various Plastics

**Figure 6:**
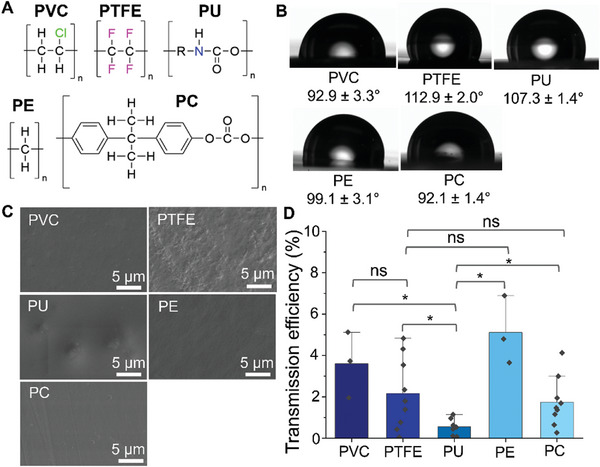
Transmission of live *E. coli* onto various plastic coupons. A) Chemical structures of plastics used in this study. B) WCA of each plastic coupon. C) Surface structures of each plastic. D) Transmission efficiency of live bacteria on each plastic. Error bars represent the standard deviation generated from 9 replicates in three experiments for PTFE, PU, and PC, and 3 replicates in one experiment for the rest. ^*^: *p* ≥ 0.05, ns: *p* ≤ 0.05.

Next, the transmission of *E. coli* onto various everyday plastics was studied. Five plastics with different chemical compositions and structures were selected, including polyvinyl chloride (PVC), polytetrafluoroethylene (PTFE), polyurethane (PU), polyethylene (PE) and polycarbonate (PC) (**Figure** **6**A). The wettability of these plastics was all hydrophobic, ranging from 90° to 120° (Figure 6B). Most of the plastics used in this study had flat surface structures at the microscale, although some flat microstructures were observed for PTFE (Figure 6C). The transmission efficiencies of tested plastics ranged from 0.5% to 5.1% (Figure 6D). Interestingly, PU demonstrated a significantly lower transmission efficiency compared to others including PTFE.

### 2.4 Transmission Efficiency of Live Bacteria onto Antimicrobial Surfaces

**Figure 7:**
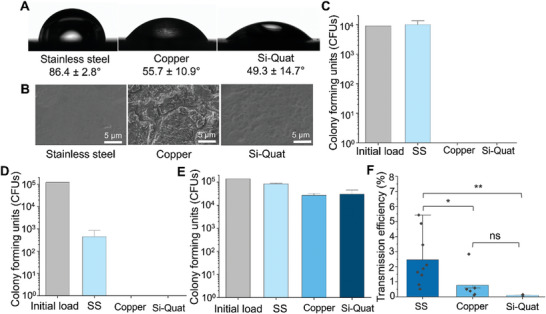
Antimicrobial efficacy and transmission efficiency of stainless steel (SS), copper, and Si‐Quat coating for *E. coli*. A) WCA of each coupon. B) Surface structures of each coupon. C) CFUs quantified after wet antimicrobial testing (modified ISO22196 with an incubation time of 2 h). The initial load indicates the number of CFUs in the bacterial suspension used for testing. D) CFUs quantified after antimicrobial testing under the dry condition with an incubation time of 2 h. E) CFUs quantified after the HydroTouch test. F) Transmission efficiency of live bacteria calculated from the HydroTouch testing result. Error bars represent the standard deviation generated from three replicates in one experiment for C, D, and Si‐Quat in F, and from nine and six replicates in three and two experiments for SS and Copper, respectively, in F. ^*^: *p* ≤ 0.05, ^**^: *p* ≤ 0.01, ns: 0.05 ≥ *p*.

Lastly, the HydroTouch test was employed to quantify the antimicrobial efficacy and transmission efficiencies of coupons with previously known antimicrobial effects.^[^ ^8^, ^9^ ^]^ A copper sheet and stainless steel coated with a commercial antimicrobial spray containing silica‐modified quaternary ammonium as the active ingredient (Si‐Quat) were used. Both antimicrobial coupons were relatively hydrophilic compared to stainless steel (**Figure** **7**A). The stainless steel coupons, with and without the Si‐Quat coating, were found to be flat at the microscale, whereas the copper exhibited a notably rough texture (Figure 7B). The antimicrobial efficacy of each antimicrobial coupon was initially confirmed using the ISO 22196 testing with modifications, where the bacterial suspensions were in contact with each coupon for 2 h. The results demonstrated strong antimicrobial activities for both the copper sheet and the Si‐Quat coating, with no CFUs detected after 2 h incubation of bacterial suspension in contact with each coupon (Figure 7C). In contrast, the number of CFUs on stainless steel remained unchanged compared to the initial loading.

Next, the antimicrobial efficacy was further assessed using a dry test. In the dry test, bacterial suspensions were applied to each coupon, and allowed to dry completely following a previously established protocol.^[^ ^10^, ^26^ ^]^ After 2 h incubation on each coupon, no CFUs were retrieved from copper and Si‐Quat coupons, similar to the wet test results (Figure 7D). However, unlike the wet test, the number of CFUs recovered from stainless steel dramatically reduced compared to the initial load. In the HydroTouch test, a coupon was in contact with bacteria on a gelatin pad for 10 s followed by immediate bacterial recovery from the gelatin pad and incubation in the dry condition (air) for 7 min before recovery from the coupon. Under these test conditions, stainless steel exhibited only a slight reduction in CFUs. Copper and Si‐Quat coatings showed a higher reduction in CFUs than stainless steel but a lower reduction compared to the results in wet or dry testing (Figure 7D). The calculated antimicrobial efficacies for stainless steel, copper, and Si‐Quat coatings are 12.1%, 68.6%, and 70.3%, respectively. Finally, transmission efficiencies were calculated, confirming significantly lower transmission of living bacteria onto the two antimicrobial coupons compared to stainless steel, with no significant differences between them (Figure 7F).

## 3 Discussion

Transmission of pathogens via hands and environmental surfaces is a critical route for HAIs. Most studies on transmission have focused on human fingers, where volunteers are trained to press coupons with a defined load. Our research demonstrated variability in creating a similar level of touching pressure between individuals and even between fingers of the same person. Additionally, direct swabbing, a common method for collecting bacteria from finger skin post‐testing, can complicate the quantitative determination of transmission efficiencies due to variability in swabbing areas and inconsistency in swabbing efficiency for micro‐structured fingertips.^[^ ^27^, ^28^ ^]^ Overall, quantitatively assessing microbial transmission via surfaces is extremely challenging, often resulting in high variability in results.^[^ ^12^, ^16^, ^24^ ^]^ It is difficult to single out the most influential factors among many, such as weight load, fingertip surface area, structure, and surface composition.

The present study demonstrates a novel method based on hydrogel to evaluate transmission efficiencies of pathogens and the antimicrobial efficacy of surfaces under realistic conditions, where a finger touches the surface with a light load for a short duration. Gelatin was chosen as an alternative to human fingers for testing due to its similarity to skin and also ease of recovery. Indeed, it was demonstrated here that gelatin resembles skin in several key aspects. The contact angle of the gelatin was 77.0 ± 2.1° after 30 min of air drying. This WCA falls within the reported range for human fingers, which varies from 60° to 120° depending on treatment.^[^ ^25^ ^]^ The pH of normal skin is acidic, ≈4.4–5.5, but it can vary widely due to soiling.^[^ ^29^ ^]^ The WCA of the gelatin hydrogels in buffer solutions at pH 5.5, 6.5, and 7.4 decreased compared to the hydrogel prepared in distilled water. This slight decrease is attributed to the presence of charged buffer molecules rather than the pH effect. This is evident as the gelatin hydrogel prepared using water had a pH close to 5.5 but exhibited a significantly higher WCA compared to the hydrogel in the pH 5.5 buffer, whereas the hydrogels in buffer solutions with different pH levels did not show differences in WCA. Both MES (pKa = 6.05)^[^ ^30^ ^]^ and phosphate groups (pKa1 = 2.1, pKa2 = 7.2)^[^ ^31^ ^]^ in buffer solutions are expected to exist as charged molecules at the tested pH ranges, which potentially increases the polarity of the surface, resulting in increased hydrophilicity and thus decreased WCA.^[^ ^32^ ^]^ Still, all measured WCA values are within the range of human fingers. Additionally, the gelatin hydrogel maintained its small surface porosity, not allowing for bacterial penetration into the hydrogel at all tested pH ranges, which is essential for the HydroTouch test. Moreover, it was possible to generate fingerprint‐like structures on gelatin using a negative template made from a human finger, with a resolution of 100–500 µm. Additionally, the chemical composition of gelatin pads, which includes water and proteins, makes them more similar to human skin compared to other materials used in touch transfer tests, such as PDMS and textiles. Human skin is primarily composed of water (≈65%) and proteins, with collagen being a major component (≈75% of the skin's dry weight).^[^ ^33^, ^34^, ^35^ ^]^ The skin also contains fatty acids, glycosaminoglycans (GAGs), and minerals. Therefore, the composition of gelatin pads can be further adjusted by adding these components to the gelatin gels to better mimic real skin.

The recovery of bacteria from gelatin via thermoresponsive sol–gel transition was reliable. The difference between the initial inoculum and recovered numbers could be explained by losses during handling including pipetting and spreading. Furthermore, we confirmed that gelatin, a hydrogel, offers a semi‐dry condition where bacterial survival was not affected by a drying time of up to 60 min. On a PDMS surface, which is dry, the recovery was significantly reduced after 60 min of drying compared to 30 min. This is most likely due to bacterial death by desiccation. Overall, gelatin has been demonstrated to be an effective and tunable material for touch transfer tests under highly controlled conditions, offering a reliable alternative to human fingers.

The transmission efficiency of *E. coli* from gelatin to stainless steel in our study was calculated to be 2.5 ± 1.7%. This is comparable to the reported value of 5.59 ± 4.55% for human fingers using a gram‐negative bacterium, *A. baumannii*.^[^ ^16^ ^]^ Our test results for stainless steel fluctuated within a smaller range of 0.5–5.4% (9 repeats in 3 experiments, *E. coli*) compared to the study, where the range was 0.67% to 16.70% (10 repeats),^[^ ^16^ ^]^ demonstrating the reliability of the HydroTouch test. The transmission efficiency of MRSA onto stainless steel was significantly higher than the two gram‐negative bacteria, *E. coli* and *P. aeruginosa*. A possible explanation is the relatively higher amounts and density of proteins present on the surface of Gram‐positive MRSA,^[^ ^36^, ^37^ ^]^ which can facilitate the binding of the bacteria to stainless steel via physisorption and possibly chemisorption within a short time.^[^ ^38^ ^]^ Note that in our testing conditions, the contact between bacteria and the surface was very short (10 s), which is not sufficient to allow for bacterial adhesion via biological processes. Furthermore, *P. aeruginosa* demonstrated a notably low transmission efficiency onto stainless steel. It was verified that this low transmission was not due to cell death by desiccation or loss during handling, as the total number of *P. aeruginosa* CFUs recovered from both the gelatin pad and the coupon after testing was comparable to that of *E. coli*. Further studies of bacterial attachment under conditions relevant to the HydroTouch will provide better insights to understand these results.

The observed low transmission of *E. coli* onto PU is possibly attributed to the relatively low wettability of this surface compared to others. Generally, the adsorption of proteins and cells is affected by surface wettability, with a WCA of 60–70° being optimal.^[^ ^39^ ^]^ Low wettability can provide short‐term repellency to protein/cell adsorption by minimizing interactions between the surface and proteins.^[^ ^40^ ^]^ However, the transmission efficiency of PTFE was significantly higher than that of PU and comparable to other plastics despite the highest WCA among tested plastics. This may be attributed to the relatively rough surface structures of the PTFE coupons used in this study (Figure 6C), which are known to significantly influence cell and protein attachment.^[^ ^41^ ^]^

To compare the antimicrobial activities measured by the HydroTouch test with those obtained using common antimicrobial testing methods in wet or dry conditions, stainless steel, copper, and quaternary ammonium‐based surface coatings were tested. Stainless steel, known for its lack of inherent antimicrobial properties, was chosen as a non‐antimicrobial reference. The copper surface is expected to exhibit high antimicrobial activity primarily due to the release of copper ions, which kill bacteria through several mechanisms, including disruption of cell membranes and generation of reactive oxygen species (ROS). These ROS lead to oxidative damage in RNA, proteins, and enzymes essential for microbial survival.^[^ ^42^ ^]^ The quaternary ammonium‐based surface coating consists of quaternary ammonium compounds (QACs), which are known to kill bacteria primarily by disrupting the cell membrane. This disruption occurs via strong electrostatic and hydrophobic interactions with the bacterial membrane, leading to leakage of intracellular proteins, nucleic acids, and other substances, ultimately causing cell death.^[^ ^43^ ^]^ In the modified ISO22196 test, the stainless steel showed no antimicrobial activity aligning with expectations. In the test under the dry condition, however, the number of CFU was significantly reduced after 2 h of bacterial incubation on stainless steel. This reduction is attributed to cell death due to desiccation and insufficient recovery efficiency during swabbing, which is a limitation of this method.^[^ ^19^, ^28^ ^]^ In the HydroTouch test, the calculated antimicrobial efficacy of stainless steel was ≈12%, presumably due to bacterial loss during handling rather than desiccation. This is supported by our demonstration that the number of CFU remains unaffected by a drying time of up to 60 min on gelatin (Figure 3D). Additionally, the drying time on stainless steel was 7 min, much shorter than the condition used for the dry test, resulting in less cell death by desiccation. For the copper and Si‐Quat coating, no colonies were retrieved after the wet and dry testing, whereas in the HydroTouch test, the reduction was <1 log. The HydroTouch test measures antimicrobial efficacy under different conditions compared to the wet and dry tests, not only in terms of a semi‐dry environment but also because bacteria are in contact with the antimicrobial surface for a short time. In this test, a gelatin pad, mimicking a contaminated finger, is in contact with a coupon for only 10 s. The bacteria transmitted to the coupon are then incubated in dry conditions for a defined time, 7 min in our demonstration. The HydroTouch test measures the reduction of CFUs on both the coupon and the gelatin pad induced by this short contact. This simulates the scenario of a microbially contaminated finger touching a surface. The incubation time for coupons can be adjusted to suit the purpose of the testing, mimicking real‐life scenarios where an environmental surface with transmitted bacteria can be touched again by another person at any time. Consequently, the HydroTouch test allows us to evaluate antimicrobial efficacy in this context. It was demonstrated that both copper and Si‐Quat coatings efficiently reduce the transmission of live bacteria compared to bare stainless steel. It is important to note that the calculated transmission efficiency only accounts for live bacteria. To clearly distinguish between the transfer of bacteria from gelatin to the surface and bacterial killing by the antimicrobial surface, the HydroTouch test can be combined with microscopic imaging to quantify dead bacteria on the surface.

## 4 Conclusion

The HydroTouch test has been established to quantitatively assess bacterial transmission from a finger mimetic onto environmental surfaces under conditions closely mimicking real‐life scenarios. This test demonstrated that MRSA is transmitted from fingers to stainless steel at a significantly higher efficiency (≈16%) than *E. coli* or *P. aeruginosa*. The transmission efficiency of *P. aeruginosa* was measured to be below 0.01%, the lowest among the three tested bacteria. Among various plastics, polyurethane exhibited high resistance to transmission of *E. coli*, whereas PTFE, a common water‐repellent plastic, showed a transmission efficiency of ≈2.2% comparable to stainless steel. The two antimicrobial surfaces, copper sheet and quaternary ammonium coating on stainless steel exhibited ≈10 times lower transmission efficiencies compared to stainless steel. The HydroTouch test provides an effective tier 2 method to predict antimicrobial efficacy under conditions close to real‐life environments.

## 5 Experimental Section

### Materials

Gelatin from porcine skin (Sigma‐Aldrich, G2500), plate count (PC) agar (Merck, 70152), agar (Merck, 05039), tryptic soy broth (TSB)(Merck, 22092), peptone from casein (Merck, 82303), brain heart infusion broth (BHI)(Merck, 53286), polysorbate 80 (Sigma, P4780), sodium chloride (Sigma‐Aldrich, S9888), D‐glucose (Carl Roth, 49150), meat extract (Cark Roth, X975.2), LB‐medium Lennox (Carl Roth, X964.4), lecithin (Carl Roth, AE81.1), silicone elastomer kit (Dow Corning, Sylgard 184), pH indicator (Supleco, 1.09564.0003), double‐tipped cotton swabs (100% cotton, primella wattestäbchen, Migros) and stainless steel (304 bright annealed finish) were purchased from local suppliers and used as received. The copper coupons (1 cm × 1 cm) were treated with 28% ammonia for >1 h to remove the oxide layer before use. The SiQuat coating on stainless steel was prepared by spraying a commercial product (SiQuat active coating, Affix) onto stainless steel coupons (1 cm × 1 cm) followed by air‐drying. PVC (article No.8391643), PP (article No.20689368), and PE (article No.8053649) were purchased from a local office supplier (digitec.ch). ABS (ABS plastic board white, thickness 2 mm), PTFE (PTFE film plate high‐temperature resistance sheet, thickness 0.3 mm), PC (clear PC plastic cutting plates, thickness 0.3 cm) and PU (20 × 20 inch PU plate, thickness 1 mm) were purchased from Amazon and cut into 1 cm × 1 cm coupons at the Empa St.Gallen workshop. *E. coli* ATCC 8739, *P. aeruginosa* ATCC 15442, *S. epidermis* ATCC155, *L. lactis*, DMZ4366, and *S. aureus* A1 *mec*A (MRSA) were used for experiments.

### Monitoring of Weight Loading

Prior to the experiment informed written consent from all participants was obtained. The experiment does not involve any sensitive information or pose any risks to the participants and therefore does not require approval. Two volunteers were trained to load 50 g on a scale for 10 s. During the experiment, each participant pressed on the scale with the index, middle, and ring fingers of their right hand. The holder was placed on the scale at 0 s and removed at 10 s. All experiments were recorded using an iPhone, and the weight was manually read every second.

### Preparation of Bacterial Suspension

A bacterial colony was picked from the stock culture on PC‐agar plates using an inoculating loop and transferred to 5 mL of culture medium in a 15 mL cultivation tube. For *E. coli*, 30% TSB medium supplemented with 0.25% glucose was used, while BHI medium was used for *S. epidermidis* and *L. lactis*. The tube was incubated overnight at 37 °C and 160 rpm. The overnight bacterial suspension was then diluted with the appropriate medium for each test.

### Preparation and Characterization of Gelatin Pads

To prepare gelatin gels, 50 mL of dw was added to 5 g of gelatin in a 100 mL bottle with a cap. For preparing gelatin gels at varying pH levels, 50 mm PBS buffer at pH 6.5 or 7.4, and 50 mm MES (2‐(N‐morpholino)ethanesulfonic acid) buffer at pH 5.5 were used instead of dw. The bottle was loosely capped and placed in an oil bath at 60 °C, with the solution stirred at 200 rpm for 20 min. The gelatin solution was then sterilized by filtration using a 0.22 µm filter. For gelation, 20 mL of the solution was transferred to a Petri dish (90 mm in diameter) and cooled at RT for 1 h. For storage, the dishes with gelatin gels were sealed with parafilm and stored upside down at 4 °C. To prepare the test pads for the touch transfer test, the gel prepared in the Petri dish was cut into square pieces measuring 1.1 cm × 1.1 cm using a custom‐made cutting device.

WCA was measured using the optical contact angle measuring and contour analysis system equipped with a humidity controller (Dataphysics) at 40% relative humidity. For each measurement, a 2 µL water droplet was placed on the surface, and a picture was taken after 5 s to measure the WCA. For each coupon, three to five measurements for triplicates were conducted. For SEM analysis, the gelatin gels were frozen at −80 °C and then lyophilized. A Pd/Au coating (7.5 nm) was applied to the gels before analysis with SEM (Axia ChemiSEM, ThermoFisher Scientific).

For the penetration test, gelatin gels (1 g or 5 g gelatin in 50 mL dw) containing culture media (0.5 g LB for *E. coli*, 0.5 g BHI for *S. epidermis* and *L. lactis*) were prepared. A 10 µL bacterial suspension (OD_600_ = 0.125) was spread on the gel using an inoculating loop and dried at RT for 20 min. To prepare the positive control, the bacterial suspension was added to the gelatin solution at ≈37 °C, gently mixed, and allowed to cool for gelation. All plates were then incubated at RT for 1 day for *E. coli* and for 5 days for *S. epidermidis* and *L. lactis*. Pictures were taken using Scan 300 colony counter (Interscience).

The gelatin gel with the fingerprint structure was prepared according to a reported method.^[^ ^44^ ^]^ Briefly, 0.5 mL of acetone was applied to the PC surface to cover an area of ≈1.5 cm × 1.5 cm. After 45 s, the acetone was removed by gently tilting the surface, immediately followed by pressing an index finger on the acetone‐treated area for 1–3 s. The negative fingerprint was surrounded by PDMS gel (3 mm thick, 1:40) to form a well, into which the gelatin solution was poured, and a glass slide was placed on the gelatin solution. The gelatin was cooled to form a gel and then removed from the PC template. The gels were analyzed by optical microscopy (Primovert inverted microscope, ZEISS) and SEM after lyophilization.

### Optimization of Washing Method

To test different washing methods for the recovery of bacteria from coupons, stainless steel coupons were used. The coupon was pressed with a gelatin pad loaded with E. coli (5 log10 CFU) for 10 s. Afterward, the coupons were collected in a 15 mL tube containing 2 mL PBS, followed by vortexing for 30 s or sonication for 5 min in a sonication bath (Sonorex Super RK 1029H, Bandelin). For swabbing, a double‐tipped cotton swab was used. The coupon was first cleaned with one tip pre‐wetted with 100 µL PBS, followed by a second cleaning using the other dry tip. Both tips were collected in a 15 mL tube containing 2 mL PBS by cutting the tips with scissors. The presence of residual bacteria on the cleaned coupons was confirmed by pouring ≈20 mL of PC‐agar solution at 40 °C onto the coupon, allowing it to gel at RT for 1 h, and then incubating it at 37 °C overnight.

### HydroTouch Test

A 1:500 diluted nutrient broth (NB) was prepared according to ISO 22196 and used as the suspension medium. The bacterial suspension at 7 log10 CFU was prepared by diluting the overnight pre‐cultured solution with the suspension medium. The suspension was then kept on ice at 4 °C until the end of the test. In a sterile bench, a gelatin pad (1.1 cm × 1.1 cm) was placed in the middle of a holder using tweezers. A 5 µL bacterial suspension was added to the surface of the gelatin pad and carefully spread using an inoculating loop, followed by drying at RT for 30 min. Meanwhile, the coupons to be tested (1 cm × 1 cm) were placed in triplicate on a PDMS (base elastomer: catalyst (wt/wt) = 1:40)‐coated Petri dish (90 mm). The PDMS coating was used to hold the test coupon in place upon detachment of the gelatin pad. After 30 min drying of the 5 µL cell suspension, the holder with the gelatin pad was placed on top of a test coupon and kept for 10 s, followed by immediate removal by lifting the holder. The coupon was incubated at RT for 7 min before swabbing.

The gelatin pad was collected in a 12‐well plate containing 1 mL of a neutralizing dissolution medium, PBS pH 7.4 containing lecithin (0.1 wt%) and polysorbate 80 (0.7 wt%) as neutralizers. The well plate with gelatin pads was then incubated at 37 °C and 150 rpm to allow sol‐transition of the gelatin gel. After 20 min, the dissolved gelatin was serially diluted using PBS until reaching a dilution of 10–3. 20 µL of each diluted solution was spotted on PC agar plates in triplicates.

For washing the tested coupons, soybean casein digest broth containing lecithin and polysorbate 80 (SCDLP), prepared according to ISO22196, was used as the neutralization and washing medium. The coupon was swabbed once with a tip pre‐wetted with 100 µL SCDLP, followed by a second swabbing with the dry tip of a double‐tipped cotton swab. Both tips were collected in a 15 mL flat‐bottom tube containing 2 mL SCDLP by cutting the tips with sterile scissors. The tube was vortexed for 30 s, and then 200 µL of the solution was collected for serial dilution with PBS until reaching 10^−3^. 20 µL of each diluted solution was spotted on PC agar plates in triplicates. All plates were incubated at 37 °C overnight, followed by colony counting.

### Wet and Dry Antimicrobial Test

The antimicrobial test under wet conditions was conducted according to a modified ISO 22196 protocol. Briefly, the bacterial suspension was prepared by diluting the overnight pre‐culture with the suspension medium (1:500 diluted NB) to achieve a concentration of 7 log10 CFU mL^−1^. A total of 50 µL of the suspension was placed on each coupon to achieve a final density of 5 log10 CFU per coupon. The surface was then covered with a cover slide to prevent drying and incubated at RT for 2 h in a plastic container with wet paper. Afterward, the coupon was transferred to a 15 mL tube containing 2 mL of SCDLP and vortexed for 30 s. The washing solution was serially diluted using PBS to a dilution of 10–3. 20 µL of each diluted solution was spotted on PC agar plates in triplicate, followed by overnight incubation at 37 °C. Colonies were then counted.

The antimicrobial test under dry conditions was performed following the protocol by Hirsch et al. with modifications.^[^ ^26^ ^]^ The bacterial suspension was prepared using the same method as the wet test but adjusted to achieve a concentration of 8 log10 CFU mL^−1^. Four drops of the suspension (1.5 µL per drop) were placed on each coupon and incubated at RT and relative humidity of ≈45% for 2 h. Afterward, the coupons were cleaned with a double‐tipped cotton swab, following the same washing method used for the HydroTouch test. The collected solution was serially diluted with PBS to a dilution of 10^−3^. 20 µL of each diluted solution was spotted on PC agar plates in triplicate, followed by overnight incubation at 37 °C. Colonies were then counted.

### Statistical Analyses

Statistical significance was assessed using a two‐sample t‐test with Welch's correction when necessary, without pre‐processing the data, utilizing Origin software (OriginLab, USA). A *p*‐value <0.05 was considered statistically significant. Quantitative values were presented as one standard deviation from the mean. The sample size (n) for each statistical analysis ranged from 3 to 9 and was specified in the figure legends.

## Conflict of Interest

The authors declare no conflict of interest.

## Supporting information



Supporting Information

## Data Availability

The data that support the findings of this study are available from the corresponding author upon reasonable request.

## References

[1] D. J. Weber , W. A. Rutala , Infec. Cont. Hos. Epidemiol. Am. 2013, 34, 449. 10.1086/670223 23571359

[2] J. M. Boyce , J. Hosp. Infect. 2007, 65, 50. 17540242 10.1016/S0195-6701(07)60015-2

[3] R. A. Weinstein , Am. J. Med. 1991, 91, S179.

[4] J. A. Otter , S. Yezli , G. L. French , Infect. Cont. Hosp. Ep. 2011, 32, 687. 10.1086/660363 21666400

[5] S. A. Sattar , J. Y. Maillard , Am. J. Infect. Control. 2013, 41, S97. 23622759 10.1016/j.ajic.2012.10.032

[6] K. Huslage , W. A. Rutala , M. F. Gergen , E. E. Sickbert‐Bennett , D. J. Weber , Infect. Cont. Hosp. Ep. 2013, 34, 211. 10.1086/669092 23295570

[7] W. A. Rutala , D. J. Weber , Infect. Dis. Clin. N. Am. 2011, 25, 45. 10.1016/j.idc.2010.11.009 21315994

[8] M. G.‐N. A Jose , S. Swift , Appl. Microbiol. 2023, 3, 145.

[9] M. Birkett , L. Dover , C. C. Lukose , A. W. Zia , M. M. Tambuwala , A. Serrano‐Aroca , Int. J. Mol. Sci. 2022, 23, 1162. 35163084 10.3390/ijms23031162 PMC8835042

[10] ISO 22196:2011, Measurement of antibacterial activity on plastics and other non‐porous surfaces, 2011, https://www.iso.org/standard/54431.html.

[11] ISO 7581:2023, Evaluation of bactericidal activity of a non‐porous antimicrobial surface used in a dry environment, 2023, https://www.iso.org/standard/83386.html.

[12] G. U. Lopez , C. P. Gerba , A. H. Tamimi , M. Kitajima , S. L. Maxwell , J. B. Rose , Appl. Environ. Microb. 2013, 79, 5728. 10.1128/AEM.01030-13 PMC3754157 23851098

[13] M. Ojeil , C. Jermann , J. Holah , S. P. Denyer , J. Y. Maillard , J. Hosp. Infect. 2013, 85, 274. 24091310 10.1016/j.jhin.2013.08.007

[14] P. C. Zhao , P. O. Chan , Y. S. Gao , O. W. Lai , T. Zhang , Y. G. Li , Build. Environ. 2019, 158, 28.

[15] H. Kaur , M. Rosenberg , M. Kook , D. Danilian , V. Kisand , A. Ivask , Fems. Microbes. 2024, 5, xtad022. 38213394 10.1093/femsmc/xtad022 PMC10781430

[16] C. Greene , G. Vadlamudi , M. Eisenberg , B. Foxman , J. Koopman , C. Xi , Am. J. Infect. Control 2015, 43, 928. 26141689 10.1016/j.ajic.2015.05.008 PMC10062061

[17] A. Perez‐Gavilan , J. V. de Castro , A. Arana , S. Merino , A. Retolaza , S. A. Alves , A. Francone , N. Kehagias , C. M. Sotomayor‐Torres , D. Cocina , R. Mortera , S. Crapanzano , C. J. Pelegrín , M. C. Garrigos , A. Jiménez , B. Galindo , M. C. Araque , D. Dykeman , N. M. Neves , J. M. Marimón , Sci. Rep. 2021, 11, 6675. 33758227 10.1038/s41598-021-85995-9 PMC7988007

[18] R. MacLachlan , F. Vahedi , S. M. Imani , A. A. Ashkar , T. F. Didar , L. Soleymani , ACS Appl. Mater. Interfaces 2022, 14, 11068. 35225604 10.1021/acsami.1c21476

[19] C. E. Santo , E. W. Lam , C. G. Elowsky , D. Quaranta , D. W. Domaille , C. J. Chang , G. Grass , Appl. Environ. Microb. 2011, 77, 794. 10.1128/AEM.01599-10 PMC3028699 21148701

[20] J. Esbelin , T. Santos , M. Hébraud , Food Microbiol. 2018, 69, 82. 28941912 10.1016/j.fm.2017.07.017

[21] R. Schrieber , Gareis, H. , From Collagen to Gelatine, Wiley‐VCH Verlag GmbH & Co., Weinheim, Germany, 2007.

[22] K. Su , C. M. Wang , Biotechnol. Lett. 2015, 37, 2139. 26160110 10.1007/s10529-015-1907-0

[23] X. Liu , M. J. Carré , Q. Zhang , Z. Lu , S. J. Matcher , R. Lewis , Skin Res. Technol. 2018, 24, 31. 28573767 10.1111/srt.12387

[24] M. F. King , M. López‐García , K. P. Atedoghu , N. Zhang , A. M. Wilson , M. Weterings , W. Hiwar , S. J. Dancer , C. J. Noakes , L. A. Fletcher , Indoor Air 2020, 30, 993. 32329918 10.1111/ina.12682

[25] M. E. Ginn , C. M. Noyes , E. Jungermann , J. Colloid. Interface Sci. 1968, 26, 146. 5650915 10.1016/0021-9797(68)90306-8

[26] M. D. Campos , P. C. Zucchi , A. Phung , S. N. Leonard , E. B. Hirsch , PLoS One 2016, 11, 0160728. 10.1371/journal.pone.0160728 PMC4975443 27494336

[27] K. Ogai , S. Nagase , K. Mukai , T. Iuchi , Y. Mori , M. Matsue , K. Sugitani , J. Sugama , S. Okamoto , Front. Microbiol. 2018, 9, 2362. 30333815 10.3389/fmicb.2018.02362 PMC6176111

[28] S. Keeratipibul , T. Laovittayanurak , O. Pornruangsarp , Y. Chaturongkasumrit , H. Takahashi , P. Techaruvichit , Food Control 2017, 77, 139.

[29] E. Proksch , J. Dermatol. 2018, 45, 1044. 29863755 10.1111/1346-8138.14489

[30] M. Haering , M. M. Pérez‐Madrigal , D. Kühbeck , A. Pettignano , F. Quignard , D. D. Díaz , Molecules 2015, 20, 4136. 25749682 10.3390/molecules20034136 PMC6272779

[31] L. Samuelsen , R. Holm , A. Lathuile , C. Schönbeck , Int J Pharmaceut 2019, 560, 357. 10.1016/j.ijpharm.2019.02.019 30797864

[32] S. Standal , J. Haavik , A. M. Blokhus , A. Skauge , J Petrol Sci Eng. 1999, 24, 131. 10.1006/jcis.1998.5988 10072272

[33] H. H. Mitchell , T. S. Hamilton , F. R. Steggerda , H. W. Bean , J. Biol. Chem. 1945, 158, 625.

[34] H. K. Graham , A. Eckersley , M. Ozols , K. T. Mellody , M. J. Sherratt , Stud. Mechanobiol. Tis. 2019, 22, 1.

[35] J. W. Shin , S. H. Kwon , J. Y. Choi , J. I. Na , C. H. Huh , H. R. Choi , K. C. Park , Int. J. Mol. Sci. 2019, 20, 2126.31036793

[36] V. A. Fischetti , Microbiol. Spectr. 2019, 7, 4. 10.1128/microbiolspec.gpp3-0012-2018 PMC6684298 31373270

[37] T. J. Silhavy , D. Kahne , S. Walker , Cold Spring Harb. Perspect. Biol. 2010, 2, a000414. 20452953 10.1101/cshperspect.a000414 PMC2857177

[38] Y. Hedberg , X. Wang , J. Hedberg , M. Lundin , E. Blomberg , I. O. Wallinder , J. Mater. Sci. Mater. M. 2013, 24, 1015. 23378148 10.1007/s10856-013-4859-8 PMC3620448

[39] Y. Arima , H. Iwata , Biomaterials 2007, 28, 3074. 17428532 10.1016/j.biomaterials.2007.03.013

[40] Z. K. He , X. R. Lan , Q. S. Hu , H. M. Li , L. M. Li , J. Y. Mao , Prog. Org. Coat. 2021, 157, 106285.

[41] T. Akkas , C. Citak , A. Sirkecioglu , F. S. Güner , Polym. Int. 2013, 62, 1202.

[42] I. Salah , I. P. Parkin , E. Allan , RSC Adv. 2021, 11, 18179. 35480904 10.1039/d1ra02149d PMC9033467

[43] S. T. Wang , B. W. Qiu , J. A. Shi , M. Wang , J. Coat. Technol. Res. 2024, 21, 87.

[44] C. W. Schultz , J. X. H. Wong , H. Z. Yu , Sci. Rep. 2018, 8, 9613. 29941987 10.1038/s41598-018-27885-1 PMC6018551

