# Supplementary material for: Quantitative Assessment of Microbial Transmission onto Environmental Surfaces Using Thermoresponsive Gelatin Hydrogels as a Finger Mimetic under In Situ‐Mimicking Conditions
Source: Adv Healthc Mater. 2025 Jan 15;14(6):2403790. doi: 10.1002/adhm.202403790 (PMC11874667; doi:10.1002/adhm.202403790)
Supplement: Supplementary file 1 — Supporting Information [file ADHM-14-0-s001.docx]

Supporting Information

Quantitative Assessment of Microbial Transmission onto Environmental Surfaces Using Thermoresponsive Gelatin Hydrogels as a Finger Mimetic under In situ-Mimicking Conditions

Mihyun Lee^1,^*, Luzia Wiesli^1^, Frank Schreiber^2^, Angela Ivask^3^, Qun Ren^1,^*


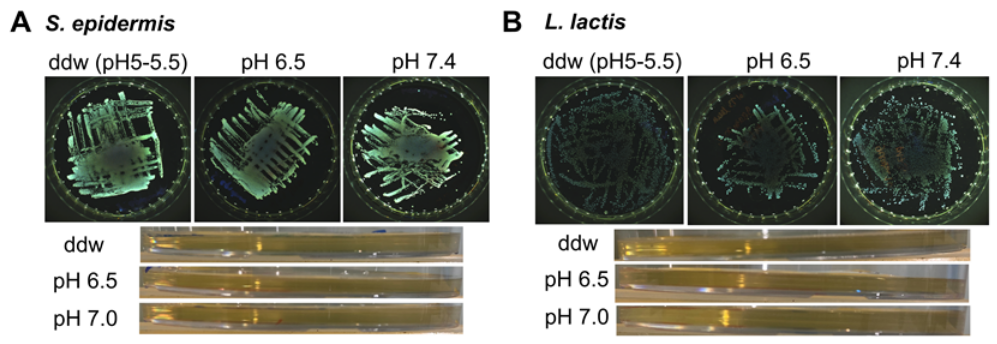


**Figure S1**. Penetration of gram-positive bacteria to gelatin gels.


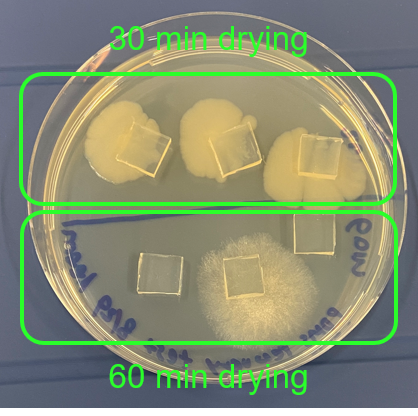


**Figure S2**. Colonial growth of *E. coli* from PDMS after swabbing embedded in a agar gel.
